# A Cost-Effectiveness Analysis of a Home-Based HIV Counselling and Testing Intervention versus the Standard (Facility Based) HIV Testing Strategy in Rural South Africa

**DOI:** 10.1371/journal.pone.0135048

**Published:** 2015-08-14

**Authors:** Hanani Tabana, Lungiswa Nkonki, Charles Hongoro, Tanya Doherty, Anna Mia Ekström, Reshma Naik, Wanga Zembe-Mkabile, Debra Jackson, Anna Thorson

**Affiliations:** 1 Health Systems Research Unit, Medical Research Council of South Africa, Cape Town, South Africa; 2 Department of Public Health Sciences, Karolinska Institutet, Stockholm, Sweden; 3 School of Public Health, University of the Western Cape, Cape Town, South Africa; 4 Department of Infectious Diseases, Karolinska University Hospital, Stockholm, Sweden; 5 Division of Community Health, Faculty of Health Sciences, Stellenbosch University, Cape Town, South Africa; 6 Department of Global Health, Boston University School of Public Health, Boston, United States of America; 7 Population Reference Bureau, 1875 Connecticut Avenue, NW Suite 520, Washington, DC, United States of America; 8 Population Health, Health Systems and Innovation, Human Sciences Research Council, Pretoria, South Africa; Emory University School of Medicine and Children's Healthcare of Atlanta, UNITED STATES

## Abstract

**Introduction:**

There is growing evidence concerning the acceptability and feasibility of home-based HIV testing. However, less is known about the cost-effectiveness of the approach yet it is a critical component to guide decisions about scaling up access to HIV testing. This study examined the cost-effectiveness of a home-based HIV testing intervention in rural South Africa.

**Methods:**

Two alternatives: clinic and home-based HIV counselling and testing were compared. Costs were analysed from a provider’s perspective for the period of January to December 2010. The outcome, HIV counselling and testing (HCT) uptake was obtained from the Good Start home-based HIV counselling and testing (HBHCT) cluster randomised control trial undertaken in KwaZulu-Natal province. Cost-effectiveness was estimated for a target population of 22,099 versus 23,864 people for intervention and control communities respectively. Average costs were calculated as the cost per client tested, while cost-effectiveness was calculated as the cost per additional client tested through HBHCT.

**Results:**

Based on effectiveness of 37% in the intervention (HBHCT) arm compared to 16% in control arm, home based testing costs US$29 compared to US$38 per person for clinic HCT. The incremental cost effectiveness per client tested using HBHCT was $19.

**Conclusions:**

HBHCT was less costly and more effective. Home-based HCT could present a cost-effective alternative for rural ‘hard to reach’ populations depending on affordability by the health system, and should be considered as part of community outreach programs.

## Introduction

Almost 30 years into the HIV epidemic, and billions of dollars spent in efforts to reduce the disease burden, research is needed on estimating costs and effectiveness of various programs to adequately inform HIV prevention[1]. Few reviews of cost effectiveness of HIV prevention interventions in low-and middle-income countries have been conducted [2,3]. HIV counselling and testing (HCT) is one of many HIV prevention strategies and remains central in the development of a response to the HIV/AIDS epidemic in sub-Saharan Africa (SSA) [4]. The commonly known and long existing strategy of HCT is facility based opt-in testing [4]. Alternative strategies to increase uptake of HCT include: mobile or community HCT, home-based testing, provider initiated counselling and testing (PITC), national HCT campaigns and integrated HCT. These strategies have had varying degrees of success in increasing testing uptake [5–7]. Home-based HCT (HBHCT) is increasing in popularity, especially for rural populations [7,8], with few randomised control trials which have rigorously assessed the effectiveness of this approach [9,10], [11]. In view of resource scarcity, the pertinent research question to ask is whether or not the strategy is cost-effective, also at a larger scale. Affordability and sustainability are key issues to the success of any widespread implementation of a health programme.

In 2010, the World Bank released a report that showed that, in the health sector, only one of the 24 HIV prevention projects that purported to conduct a cost-effectiveness analysis (CEA), in fact did so. The report concluded that CEA in health is very rare and when applied is often misconstrued as a simple cost analysis [1]. The challenge with most CEA studies conducted to date is the limited robust primary data from a single source [12].

A recent review in SSA reported that two studies conducted cost analyses of HBHCT and reported costs per client tested of less than US$9 [13]. In 2008, a Kenyan study reported a cost of $5.88 for each person tested, and $84 per positive case detected [14]. These studies were not full economic evaluations, but cost analyses. Thus, there is a dearth of evidence on the cost-effectiveness of HBHCT to inform its widespread implementation [15]. The aim of the current study was to conduct a cost-effectiveness analysis comparing home-based with clinic based HCT. We also examined costs of resources required for intervention scale-up using a South African district level structure by modelling an operational scenario.

## Materials and Methods

### Setting

The study was conducted in a rural sub-district located in KwaZulu Natal, South Africa, a province with one of the highest HIV prevalence rates (17%) in the general population [16]. A cross-sectional survey conducted in the study area indicated that only 16% of the adult population reported ever having an HIV test, often at a clinic [17]. The sub-district has a population of approximately 243,000 people, with 70% of the households living below the poverty line [18]. More details of the study setting and population are described elsewhere [19].

### Intervention (home-based HCT)

The trial design and intervention have been described fully elsewhere [20]. The trial was implemented in 16 clusters, each consisting of 100 to 200 households, with an average household size of 3 persons. The adult population in the 16 study clusters was approximately 46,000 people [21]. The intervention was door-to-door HCT offered to all adults (18 years and above, and to 14–17 year olds with guardian/parental consent) residing in the eight intervention clusters. All consenting individuals were tested for HIV. The HIV testing algorithm followed the national HIV testing guidelines and involved rapid HIV tests. A dried blood spot was taken at the same time as the rapid test for quality control purposes for all participants, then later for HIV positive participants only [20].

In the eight control areas, standard of care (facility-based HIV testing) was available. Home-based and clinic HIV testing follow similar procedures including; pre-test counselling, testing and post-test counselling between a trained counsellor and a client, couple or group of people.

Personnel who delivered the intervention and their total time spent in the project are summarised in Table 1.

**Table 1:** Good Start intervention staff.

Staff category	Number	Total time spent on project
Site project manager	1	100%
Supervisors	4	100% for 3, and 50% for 1 supervisor who also had a 50% study coordination role
Lay counsellors	11	100%
Drivers	3	100% for 2, and 50% for 1
Cleaner	1	20%

Four supervisors were employed on a full time basis to supervise eleven lay counsellors in the trial. One supervisor was assigned 2–3 lay counsellors depending on the size of the catchment area. Supervisor roles included; daily lay counsellor supervision, observational counsellor assessments at least twice a week, ‘troubleshooting’ any challenges, collection of dry blood spot samples and client exit interviews.

Eleven trained lay counsellors from the study communities delivered the intervention. Their characteristics are described elsewhere [20]. Lay counsellors completed an initial ten-day nationally accredited course in HCT and spent four months completing in-service training at local clinics. In addition, they received training on; prevention of mother-to-child HIV transmission, couples counselling, TB and STI screening, family counselling, HIV and infant feeding; disclosure; and family planning. Supervisors attended all the lay counsellor-training sessions.

### Clinic HIV counselling and testing (control arm)

HCT in the facilities is both provider and client initiated. Lay counsellors test and counsel clients, while professional nurses verify positive results and occasionally test clients. In the study control areas, HCT in clinics was integrated with other programs such as tuberculosis (TB) or sexually transmitted infections (STI) care, thus staff time was shared. Staff in the clinics included lay counsellors who conducted HIV testing, professional nurses (lay counsellor supervisors), security guards and cleaners.

### Data collection

We collected cost data for a period of 12 months (January to December 2010). The instrument for collecting data was adapted from validated costing tools [11,22] and developed in Excel. We performed retrospective reviews of expenditure records, supplemented and validated data by interviewing key personnel.

The Good Start HBHCT was a research project implemented as a vertical intervention. Resources only used for research purposes were apportioned appropriately and excluded in the analysis. Examples of these included; electronic data collection system (research console), part of the DBS costs, some stationary such as mobile phone recharge vouchers for data collection, and research staff in the home based HCT arm. The study team discussed allocation of inputs to research versus intervention costs until a consensus was reached. Intervention cost allocation is presented in Table A in S1 File.

### Lay counsellors’ time

Lay counsellors in the intervention arm completed logs of time spent in each household by capturing their start and end times. The data was validated using the electronic study database by viewing real time data submitted to a data management console. This data was collected in light of implications for incorporating home-based HCT into existing community programmes if the intervention were to be scaled up.

For the control communities, four public clinics (that offer HCT among other services) in the study population were purposefully sampled. The clinics were representative of populations served in terms of volumes of people presenting in the clinics, and distance of clinics from major roads. Methods of data collection included reviewing clinic registers, expenditure records and informal interviews with key people to supplement and validate data from records.

### Measurement of costs

The cost of the intervention was calculated using the ingredients approach [11]. All inputs were identified, measured, valued and grouped into overheads and four activities associated with the intervention as follows; (i) start up, (ii) overheads, (iii) training, (iv) HIV counselling and testing, and (v) HIV lay counsellor supervision. We broadly distinguished between start up and implementation costs (Table A in S1 File).

Start up costs were treated as capital and assigned 5 useful life years to reflect their potential use not only in program scale up but also in other settings. These included; intervention design, manuals and study materials, and community mobilisation costs. Useful life years for initial training were estimated to be 5 years based on the number of years the information acquired is likely to remain relevant or before there is new information available. Costs for the activity HIV counselling and testing were for personnel and testing equipment, all classified as recurrent, hence not annualised.

Lay counsellor supervision included costs of lay counsellor supervisors and vehicles. Costs were classified into capital and recurrent. Recurrent costs included personnel, testing equipment, vehicle maintenance and fuel costs, stationary, office rental and utilities. Capital costs included vehicles, office furniture, equipment, buildings and any items whose useful life was more than a year while capital items purchased for less than US$100 were treated as recurrent costs [11].

We used 5, 7, and 30 years for useful life years for furniture, vehicles, and buildings respectively. Annual economic costs of capital items were calculated using either a purchase value or replacement value of the item, the estimated number of useful life years, and annualized using an interest rate of 9% (the return on South African long term government bonds for 2010) [22] and deriving the corresponding annuity factor from a standard table [11], [23].

Apportionment of overhead costs in the clinic comparator was achieved through taking measurements of the whole building and rooms where HCT activities are carried out. Thereafter a ratio of HCT room to clinic was computed. The derived ratio was used to allocate costs of the HCT room, cleaners, security staff and other overheads.

The relevant costs and effects as framed by the comparison statement and viewpoint occur in the present. In other words, the analysis is conducted at one point in time and the analytic horizon of the outcome (HCT uptake) is well within 1 year.

The South African currency (Rand) was converted to the US Dollar (US$) using the average exchange rate (7.3 ZAR to 1 US$) for 2010[19]. For input costs incurred prior to 2010, we adjusted for inflation by using the consumer price index (CPI), using 2010 as a base year [24]. Market prices were used for items where the price was not available.

### Measurement of effectiveness and cost-effectiveness

The main outcome of the trial was uptake of HCT measured as an increase in HIV testing uptake from baseline (pre-intervention) to endline (post-intervention), that is, the increase in the proportion of those offered HCT and accepted to be tested at baseline compared to endline [10]. Population effectiveness was thus estimated as, percentage increase in HCT uptake multiplied by the catchment population size for each arm (HBHCT and clinic HCT). Average costs for the intervention and comparison were also calculated by dividing total annual costs by the population effectiveness. This paper reports cost-effectiveness of the intervention as the incremental cost per additional HIV test.

### Sensitivity analyses

One-way sensitivity analyses were undertaken to test the robustness of the cost-effectiveness estimates to variations of identified variables with uncertainties. These variables included; the discount rates (3% and 6%), professional nurse salaries, catchment population sizes and HIV test kits (Table B in S1 File).

### Operational scenario

In addition to comparing HBHCT with clinic HCT, we present costs of an operational scenario to demonstrate costs of implementing the intervention through integration into the district health system’s community programs run by the department of health. Critical intervention inputs were identified and relevant costs presented. Inputs were thus varied where relevant, to match the district structure (Table 2). The operational scenario is described in detail in Box 1 in S1 File.

**Table 2:** Description of inputs varied in the operational scenario.

HBHCT	Operational scenario
**Personnel**	**Personnel**
Site project manager	Facility manager (5% time)
	District manager (1% time)
Lay counsellor supervisor	Professional nurses (team leaders) would be the equivalent in the district, 12% time)
Lay counsellors	Community health workers (12% time)
Drivers	Excluded
**Start-up costs**	**Start-up costs**
One off	Excluded
Repeatable	Repeatable
**HIV counselling and testing**	**HIV counselling and testing**
Dried blood spot samples for quality assurance (5% samples)	Excluded

### Ethical consideration

The study received ethical approval (reference number ECO12-8/2011) from the South African Medical Research Council. For this cost-effectiveness analysis, there was no data collected on individuals, thus neither verbal nor written consent was applicable. The ethics application was deemed necessary for the purposes of accessing clinic financial records and data on testing uptake.

## Results

### Effectiveness

Comparisons between baseline and post-intervention showed that HIV testing uptake increased by 37% (from 32% to 69%) in the intervention and 16% (from 31% to 47%) in the control arm (prevalence ratio 1.54, 95% confidence interval 1.32 to 1.81) [10].

The total annual cost of implementing the vertical HBHCT intervention was US$233,239 while the clinic HCT total annual cost was US$146,615 (Table 3).

**Table 3:** Economic costs of inputs.

	HBHCT costs (US$)	% Cost within inputs	% Of total cost	Clinic HCT costs (US$)	% Cost within inputs	% Of total cost
**(i) Start up**						
One off	4 726.82	60%		-		
Repeatable	3207.38	40%		639.97	100%	
*Sub total*	*7934.20*	*100%*	*3%*	*639.97*	*100%*	* *0.4*
**(ii) Overheads**						
Office rentals/building	4182.81	86%		7851.18	68%	
Personnel (cleaners, security guards)	665.75	14%		3715.69	32%	
*Sub total*	*4848.57*	*100%*	*2%*	*11566.87*	*100%*	*8%*
**(iii) On-going training (excludes start up)**	**1797.95**	**100%**	**1%**	**6049.29**	**100%**	**4%**
**(iv) HIV counselling and testing**						
Personnel	75746.47	86%		104313.07	88%	
Testing equipment	6300.32	7%		10394.36	9%	
Stationary	3545.36	4%		3287.67	3%	
Field materials	642.01	1%		-		
Dry blood spot (DBS)	1389.63	2%				
*Sub total*	*87623.78*	*100%*	*38%*	*117995.11*	*100%*	*80%*
**(v) Lay counsellor (HCT) supervision**						
Personnel	99830.85	76%		7999.68	77%	
Vehicles	21254.77	16%		-		
Data console monthly hosting	6218.20	5%		-		
Office furniture and equipment	3730.71	3%		2364.19	23%	
*Sub total*	*131034.52*	*100%*	*56%*	*10363.87*	*100%*	*7%*
**Total in US$, (1US$: SAR7.3 in 2010)**	**233239.02**			**146615.12**		

* Percentage rounded off less than 1%

The costs per client were US$29 for HBHCT intervention and US$38 for clinic HCT (Table 4). Overall, the HBHCT intervention was more cost effective compared to clinic HCT. Consequently, the incremental cost effectiveness ratio (ICER) was US$19 per additional client tested (Table 4).

**Table 4:** Average costs, outputs and incremental cost-effectiveness ratio (ICER).

	HBHCT intervention	Clinic HCT
**Target population**	22 099	23 864
**Increase in uptake (%)**	37	16
**Effectiveness (% increase in uptake x target population)**	8177	3818
**Total annual costs (US$)**	233 239.02	146 615.12
**Cost (US$) per client (Total annual cost/effectiveness)**	29	38
**ICER (Δ costs/Δ effects)**	= ($233 239- $146 615) /(8177–3818) = 86624/4359 = $19	

### Costs

The main cost drivers for HBHCT and clinic HCT alike were: personnel salaries and office rentals in the categories; ‘HIV counselling and testing’, ‘lay counsellor supervision’ and ‘overheads’ (Table 3). In the activity HIV counselling and testing, personnel costs were slightly higher for the clinic because in addition to lay counsellors, professional nurses also tested clients yet their salary scales were much higher than those of lay counsellors. In the HBHCT, only lay counsellors conducted HIV testing.

Start up and training costs differed in their share of total costs for the clinic *versus* home-based HCT (Table 3). The differences observed in the training costs are likely due to variations in the strategies of delivering training. In the clinics, lay counsellors received a series of trainings on different topics whereas in the HBHCT, trainings were often combined. For example, training on couple counselling, TB and STIs could be given in one session, resulting in a cost saving.

### Sensitivity analyses

Results of the sensitivity analysis are presented in Table B in S1 File. There was no difference in the average costs and ICER after varying costs of the presented inputs.

### Estimating costs of the operational scenario

Table 5 compares costs of standard care, clinic HCT with the operational scenario. The modelled operational scenario demonstrated a 90% reduction in annual costs compared to the HBHCT intervention. Compared to clinic HCT, the annual cost of the operational scenario was six times less.

**Table 5:** Comparison of costs between clinic HCT and operational scenario.

	Operational scenario costs (US$) per 7,660 people	% Cost within inputs	% Of total cost	Clinic HCT costs (US$) per 23,864 people	% Cost within inputs	% Of total cost
**(i) Start up**						
One off	-	0%		-		
Repeatable	1248.59	100%		639.97	100%	
*Sub total*	*1248.59*	*100%*	*5%*	*639.97*	*100%*	* *0.4*
**(ii) Overheads**						
Office rentals/building	572.05	100%		7851.18	68%	
Personnel (cleaners, security guards)	-	0%		3715.69	32%	
*Sub total*	*572.05*	*100%*	*2%*	*11566.87*	*100%*	*8%*
**(iii) Training (excludes start up)**	**1 797.95**	**100%**	**7%**	**6049.29**	**100%**	**4%**
**(iv) HIV counselling and testing**						
Personnel	9863.01	70%		104313.07	88%	
Testing equipment	1602.64	11%		10394.36	9%	
Stationary	2559.33	18%		3287.67	3%	
*Sub total*	*14024.99*	*100%*	*57%*	*117995.11*	*100%*	*80%*
**(v) Lay counsellor (HCT) supervision**						
Personnel	3794.68	56%		7999.68	77%	
Vehicles	2812.56	41%		-		
Office furniture and equipment	179.43	3%		2364.19	23%	
*Sub total*	*6786.67*	*100%*	*28%*	*10363.87*	*100%*	*7%*
**Total in US$, (1US$: SAR7.3 in 2010)**	**24430.25**			**146615.12**		

* Percentage rounded off less than 1%

Cost drivers in the operational scenario were similar to those described in the intervention. Start up costs accounted for 5% of the total cost in the operational scenario compared to less than 1% in clinic HCT.

Overhead costs were reduced with the shift from the vertical HBHCT to an integrated community programme in the operational scenario. As a consequence, in a real setting, the integration of the intervention with existing community programmes would entail less use of office space (since community health workers would spend most of the time in the field), and related overheads, thus a reduction in costs. As a percentage share, overheads still accounted for 2% and 8% of total costs in the operational scenario and clinic HCT respectively.

On-going training costs were maintained for both approaches, though in the operational scenario, training contributed 7% of the total costs, while training in clinic HCT accounted for 4% of the total cost.

The activity HIV counselling contributed 57% towards the total cost, a percentage lower than the 80% share in clinic HCT.

Supervision of lay counsellors by professional nurses accounted for 28% and 7% of the total costs for the operational scenario and clinic HCT respectively. The biggest variation in costs for lay counsellor supervision and HIV testing activities were once again in the personnel costs for the same reasons discussed earlier. However, both salary costs were reduced in the operational scenario as community health workers who would do the testing are paid very low stipends (about $100 per month) in the South African public health sector. Further, for supervision, costs of one professional nurse supervisor are presented for the operational scenario since the modelled scenario estimates costs for one PHC outreach team as described in Box 1 in S1 File.

## Discussion

Our study shows that home-based HCT was more cost-effective in increasing uptake of HCT than clinic based HCT in this rural, high HIV-prevalence setting. The average cost per client was lower for HBHCT compared to the clinic, $29 and $38 respectively.

HIV testing forms an integral part in rolling out antiretroviral therapy (ART), treatment as prevention (TaSP) and other preventive and treatment related interventions against HIV infection. Home-based HCT would particularly form an important part of prevention strategies in high HIV prevalence areas such as KwaZulu Natal. The effectiveness of home-based HCT for rural populations has been documented in previous studies [5,9,10,25]. One of the reasons why implementation of effective programs is not happening at a big enough scale is due to scarcity of evidence on cost-effectiveness of HIV prevention programs. A cost-effectiveness study of clinic HCT by Sweat et al. 2000 reported per client costs of US$29 in Tanzania and US$27 in Kenya [26]. In Uganda, Menzies et al. 2009 reported per client costs of US$8.29 and US$19.26 for door-to-door and clinic HCT respectively [27], while in another recent study in the same country, the cost per client tested was US$6.4 for facility based HCT and US$5.0 for home based HCT [28]. Mulogo et al. 2013 further reported costs per positive case identified as US$86.5 and US$54.7 for the facility and home based HCT respectively [28]. It is difficult to compare results across studies due to different parameters and assumptions used in calculating costs, time horizon, economic climate and contexts. The studies cited here have some similarities and differences. They were conducted in rural settings, and all were conducted in areas with relatively high HIV prevalence. The differences were in the outcomes reported and populations included (while some studies focused only on adults tested, some had participants from infants to adults) [27]. Consistent with our findings, the latter studies in Kenya and Uganda, also conducted in rural settings, concluded that home-based HCT was effective in reaching more populations at a lower cost [8,27,28]. Our results highlight that different models of HCT should be considered, and home-based HCT may be recommended as an effective intervention for rural and ‘hard to reach’ (‘hard to reach’ in this context is defined as, ‘… audiences inaccessible to most traditional and conventional methods for any reason…’ as defined by Flanagan and Hancock 2010. ‘Reaching the hard to reach’—lessons learned from the VCS (voluntary and community Sector). A qualitative study. BMC Health Services Research, 10:92) populations with high HIV prevalence.

It is encouraging that the South African government has a clear commitment in investments on programs that improve health outcomes. Notably, the prominence of HIV testing in the national strategic plan (NSP), the 2011 HCT campaign, and the biggest ARV programme in the world [6] are undoubtedly commitments to increase HIV prevention efforts. Further, South Africa is currently re-engineering Primary Health Care (PHC) through use of community health workers (CHWs) to deliver health care services at home [29]. CHWs’ roles are currently limited to conducting health assessments, adherence support and basic interventions e.g. first aid [30]. HIV test finger pricking is a simple procedure, which could be introduced into the CHW scope of work with proper education, and training, as shown in our study.

Worth highlighting is that intervention impact and cost-effectiveness are not the only critical components to influence policy decision. Clearly, clinic based HCT needs to be available in addition to any home-based intervention, which implies that albeit more cost-effective, implementation would always have to be an add-on from the health system perspective. From our findings, there is no doubt that HBHCT is superior in increasing uptake of HCT and further demonstrating greater cost-effectiveness. Implementation of HBHCT into policy by governments thus depends on a number of factors, including the government’s willingness to implement such an intervention in addition to clinic HIV testing.

Supervision was a central and critical part of the intervention and it accounted for the biggest share of total costs while in the clinic arm, nurses provided supervision to lay counsellors for about 8% of their time, thus at a lower cost. However, in addition to testing done by lay counsellors in the clinic, professional nurses (higher remunerated than lay counsellors) also tested clients, hence the high personnel costs in the HIV counselling activity. Costs can be saved by task shifting duties like HIV testing to a lower cadre of workers such as CHWs/lay counsellors as recommended in previous research [31].

The operational scenario based on the current South African PHC re-engineering outreach teams structure was modelled to reflect real life setting costs. The differences in costs between the intervention (HBHCT) and operational scenario were mainly due to variations in staffing and assumed time effort. The operational scenario demonstrates that home-based HCT is a feasible and affordable intervention especially when delivered as an integrated programme. The modelled operational scenario may be used to inform program scale up. It should be noted though that in scaling up, factors such as administrative infrastructure and other critical inputs might be lacking or different in other settings hence would need to be established prior to scale up. Further, the cost of scaling up depends on the patient demand for the services offered [32]. Johns and Torres in their review on costs of scaling up health interventions also acknowledge that costs of scaling up are specific to the type of intervention and its particular setting. Planning for scale up should include, assessing capacity, availability of human resources, identifying economies and diseconomies of scale and including administrative costs [33].

There is growing research on the deployment of CHWs within the health care system in many parts of rural sub-Saharan Africa. McCord and colleagues estimated that it would take an annual average of US$3750 to train, equip and support each CHW delivering home-based care (including maternal and child health, nutrition support, HIV testing, chronic illnesses etc.) in 2015 [34]. However, there needs to be a balance of how much more CHWs can do in addition to their scope of work to ensure that the additional role does not negatively impact their performance. Previous studies have indicated that CHWs are effective when their scope of work is specific [35].

The strength of this study is that intervention effectiveness was obtained through a rigorous study design (RCT). Further, we used various sources of data including informal interviews with relevant personnel in addition to using records.

The study had some limitations. A provider perspective was used and this excludes patient costs, which may be a barrier to accessing HCT services. However, patient costs should have been minimal in the intervention since clients were tested in their homes, thereby avoiding some costs for example cost of transport to health facilities. Patient costs such as travelling expenses were not collected for the clinic HCT either. Thus, the presented costs for each alternative would probably look different if patient costs were included. Further, the costing exercise was retrospective and since we conducted informal interviews with different personnel to gather information on some resources that had been used a year ago, we cannot rule out recall bias. In addition, we did not collect individual cost data, and demographic characteristics of participants/clients in the clinic arm and thus could not report or compare these. We also did not document any differences in services offered in the different clinics that might otherwise impact access to HIV testing services. However, all clinics offered HIV testing with most of them integrating HIV testing to other programs such as antenatal care and TB. We report findings on an immediate outcome, ‘increase in HCT uptake’. While final outcomes such as HIV infections averted, disability adjusted life years (DALYs), would be ideal, these data were not measured in the trial. Finally, it would have been insightful to distinguish between initial and repeat tests to measure the effect of each strategy (and associated costs thereof) in identifying first time testers.

## Conclusions

This study demonstrated that home-based HCT was more cost-effective than clinic HCT. HBHCT presents a suitable model for increasing uptake of HCT in rural ‘hard to reach’ populations and should be considered as part of the CHW scope of work in community programs. HIV testing is of critical importance in light of the new recommendations of treatment as prevention. Given that South Africa carries the largest burden of the HIV epidemic in the world, it is one country that should have concerted efforts to increase testing rates and people initiating on antiretrovirals (ARVs) once identified as HIV positive. The financial sustainability of the program should be considered in decision-making. Our findings may be adaptable to similar settings but differences in infrastructure, health system functioning and economic climate should be considered.

## Supporting Information

S1 File Annexe A. (DOCX)

S2 File CHEERS checklist. (PDF)

## References

[1] Galárraga O, Colchero MA, Wamai RG, Bertozzi SM (2009) HIV prevention cost-effectiveness: a systematic review. BMC Public Health 9. 10.1186/1471-2458-9-S1-S5 PMC2779507 19922689

[2] Jha P, Nagelkerke JD, Ngugi EN, Prasada Rao JV, Willbond B, et al (2001) Public health. Reducing HIV transmission in developing countries. Science 292: 224–225. 11305312 10.1126/science.1058187

[3] Thielman NM, Chu HY, Ostermann J, Itemba DK, Mgonja A, et al (2006) Cost-effectiveness of free HIV voluntary counseling and testing through a community-based AIDS service organization in Northern Tanzania. Am J Public Health 96: 114–119. 16317205 10.2105/AJPH.2004.056796 PMC1470448

[4] De Cock KM, Marum E, Mbori ND (2003) A serostatus-based approach to HIV/AIDS prevention and care in Africa. Lancet 362: 1847–1849. 14654325 10.1016/S0140-6736(03)14906-9

[5] Bateganya MH, Abdulwadud OA, Kiene SM (2007) Home-based HIV voluntary counseling and testing in developing countries. Cochrane Database Syst Rev 4. 10.1002/14651858.CD006493.pub2 17943913

[6] Mayosi BM, Lawn JE, Van Niekerk A, Bradshaw D, Abdool Karim SS, et al (2012) Health in South Africa: changes and challenges since 2009. Lancet 380: 2029–2043. 10.1016/S0140-6736(12)61814-5 23201214

[7] Matovu JKB, Makumbi FE (2007) Expanding access to voluntary HIV counselling and testing in sub-Saharan Africa: alternative approaches for improving uptake, 2001–2007. Tropical Medicine and International Health 2007 12 1315–1322. 17949401 10.1111/j.1365-3156.2007.01923.x

[8] Negin J, Wariero J, Mutuo P, Jan S, Pronyk P (2009) Feasibility, acceptability and cost of home-based HIV testing in rural Kenya. Tropical Medicine and International Health 14: 849–855. 10.1111/j.1365-3156.2009.02304.x 19552646

[9] Fylkesnes K, Siziya S (2004) A randomised trial on acceptability of voluntary HIV counseling and testing. Tropical Medicine and International Health 9: 566–572. 15117300 10.1111/j.1365-3156.2004.01231.x

[10] Doherty T, Tabana H, Jackson D, Naik R, Zembe W, et al (2013) Effect of home based HIV counselling and testing intervention in rural South Africa: cluster randomised trial. BMJ 346: 1–11. 10.1136/bmj.f3481 PMC3685510 23766483

[11] Lugada E, Levin J, Abang B (2010) Comparison of home and clinic-based HIV testing among household members of persons taking antiretroviral therapy in Uganda: results from a randomized trial. J Acquir Immune Defi c Syndr 55: 245–252. 10.1097/QAI.0b013e3181e9e069 20714273

[12] Marseille E, Morin SF, Collins C, Summers T, Coates TJ, et al (2002) Cost-Effectiveness of HIV Prevention in Developing Countries. San Francisco, CA: HIV Insite-University of California San Francisco.

[13] Sabapathy K, Van den Bergh R, Fidler S, Hayes R, Ford N (2012) Uptake of home-based voluntary HIV testing in sub-Saharan Africa: a systematic review and meta-analysis. PLoS Med 9: e1001351 10.1371/journal.pmed.1001351 23226107 PMC3514284

[14] Negin J, Wariero J, Mutuo P, Jan S, Pronyk P (2009) Feasibility, acceptability and cost of home-based HIV testing in rural Kenya. Tropical Medicine and International Health 14: 849–855. 10.1111/j.1365-3156.2009.02304.x 19552646

[15] Bateganya MH, Abdulwadud OA, Kiene SM (2007) Home-based HIV voluntary counseling and testing in developing countries. Cochrane Database Syst Rev. 4. 10.1002/14651858.CD006493.pub2 17943913

[16] Shisana O, Rehle T, Simbayi LC, Zuma K, Jooste S, et al (2014) South African National HIV Prevalence, Incidence and Behaviour Survey, 2012. Cape Town, HSRC Press. 10.2989/16085906.2016.1153491 27002359

[17] Tabana H, Doherty T, Swanevelder S, Lombard C, Jackson D, et al (2012) Knowledge of HIV status prior to a community HIV counseling and testing intervention in a rural district of South Africa: results of a community based survey. BMC Infect Dis 12. 10.1186/1471-2334-12-73 PMC3338079 22458410

[18] Umzimkhulu Local Municipality: Integrated Development plan for 2009/ 2010 financial year; 2010.

[19] Tabana H, Doherty T, Rubenson B, Jackson D, Ekström AM, et al (2013) Testing Together Challenges the Relationship’: Consequences of HIV Testing as a Couple in a High HIV Prevalence Setting in Rural South Africa. PLoS ONE 8. 10.1371/journal.pone.0066390 PMC3688905 23824067

[20] Naik R, Tabana H, Doherty T, Zembe W, Jackson D (2012) Client characteristics and acceptability of a home-based HIV counselling and testing intervention in rural South Africa. BMC Public Health 12. 10.1186/1471-2458-12-824 PMC3487909 23009202

[21] Statistics South Africa. Umzimkhulu municipality; 2011. Available: http://www.statssa.gov.za.interactive.statssa.gov.za/superweb/loadDatabase.do?db=Descriptive11.wd.

[22] Adam T, Aikins M, Evans D (2007) CostIt software 2007. World health Organization, Available: http://www.who.int/choice.

[23] Drummond MF, Sculpher MJ, Torrance GW, O’Brien BJ, Stoddart GL (2007) Methods for Economic Evaluation of Health Care Programmes Third edition Oxford University Press Oxford, UK.

[24] Costing Guidelines for HIV Prevention Strategies. Joint United Nations Programme on HIV/AIDS.UNAIDS; 2000.

[25] Tumwesigye E, Wana G, Kasasa S, Muganzi E, Nuwaha F (2010) High uptake of home-based, district-wide, HIV counseling and testing in Uganda. AIDS Patient Care STDS 24: 735–741. 10.1089/apc.2010.0096 21067357

[26] Sweat M, Gregorich S, Sangiwa G, Furlonge C, Balmer D, et al (2000) Cost-effectiveness of voluntary HIV-1 counselling and testing in reducing sexual transmission of HIV-1 in Kenya and Tanzania. Lancet 356: 113–121. 10963247 10.1016/S0140-6736(00)02447-8

[27] Menzies N, Abbang B, Wanyenze R, Nuwaha F, Mugisha B, et al (2009) The cost and effectiveness of four HIV Counseling and Testing strategies in Uganda. AIDS 23: 395–401. 10.1097/QAD.0b013e328321e40b 19114865

[28] Mulogo EM, Batwala V, Nuwaha F, Aden AS, Baine OS (2013) Cost effectiveness of facility and home based HIV voluntary counseling and testing strategies in rural Uganda. Afr Health Sci 13: 423–429. 10.4314/ahs.v13i2.32 24235945 PMC3824487

[29] Pillay Y, Baron P (2011) The implementation of PHC re-engineering in South Africa. PHASA newsletter 15 November. Available: http://www.phasa.org.za/articles/the-implementation-of-phc-re-engineering-in-southafrica. Accessed 8 February 2012.

[30] Provincial Guidelines for the Implementation of the Three Streams of PHC Re-Engineering, Department of Health, Republic of South Africa; 2011.

[31] Callaghan M, Ford N, Schneider H (2010) A systematic review of task- shifting for HIV treatment and care in Africa. Human Resources for Health 8. 10.1186/1478-4491-8-8 PMC2873343 20356363

[32] Borgdorff MW, Floyd K, Broekmans JF (2002) Interventions to reduce tuberculosis mortality and transmission in low- and middle-income countries. Bulletin of the World Health Organization 80: 217–227. 11984608 PMC2567749

[33] Johns B, Torres TT (2005) Costs of scaling up health interventions: a systematic review, on behalf of WHO-CHOICE, Health System FinancingExpenditure and Resource Allocation (EIP/FER), World Health Organization, Geneva, Switzerland Health Policy and Planning 20: 1–13. 15689425 10.1093/heapol/czi001

[34] McCord GC, Liub A, Singhc P (2012) Deployment of community health workers across rural sub-Saharan Africa: financial considerations and operational assumptions. Bull World Health Organ 91: 244–253B. 10.2471/BLT.12.109660 PMC3629450 23599547

[35] Bhattacharyya K, Winch P, LeBan K, Tien M (2001) Community Health Worker Incentives and Disincentives: How They Affect Motivation, Retention, and Sustainability. Virgian Basics II.

