# Supplementary material for: A Cost-Effectiveness Analysis of a Home-Based HIV Counselling and Testing Intervention versus the Standard (Facility Based) HIV Testing Strategy in Rural South Africa
Source: PLoS One. 2015 Aug 14;10(8):e0135048. doi: 10.1371/journal.pone.0135048 (PMC4537202; doi:10.1371/journal.pone.0135048)
Supplement: S1 File — (DOCX) [file pone.0135048.s001.docx]

**S1 File: ANNEXE A**

**Table A: classification of inputs by activity for Good Start, operational scenario and clinic**

| **Activity** | **Home-based HIV testing intervention (vertical program)** | **Operational scenario (integrated program)** | **Clinic HCT (integrated program)** |
| --- | --- | --- | --- |
| **A. START UP** |  |  |  |
| **(i) One-off** |  |  |  |
| Manual development | ✔ | ✗ | ✗ |
| Intervention design | ✔ | ✗ | ✗ |
| Console development (for data collection management & monitoring) | ✔ (30%) | ✗ | ✗ |
| **Repeatable** |  |  |  |
| Lay counsellor recruitment | ✔ | ✔ | ✔ |
| Training (National HCT training course) | ✔ | ✔ | ✔ |
| Equipment (counsellor back packs, test kit cooler bags, timer clocks) | ✔ | ✔ | ✗ |
| Community mobilization (meetings, including travel) | ✔ | ✔ | ✗ |
| **B. IMPLEMENTATION** |  |  |  |
| **(ii) Overheads** |  |  |  |
| Building /office (rentals, alarm, electricity) | ✔ (80 %) | ✔ (12% space usage for HCT activities by professional nurse) | ✔ |
| Personnel (cleaners, security guards) | ✔ (80% for cleaner) | ✗ | ✔ (cleaners, security guards at specified % time for different clinics) |
| **(iii) Training (excludes start-up training)** |  |  |  |
| Refresher trainings (PMTCT, couple counselling, TB & STI topics, monthly meetings (including meals and transport allowance)) | ✔ | ✔ (excludes meals and transport) | ✔ (excludes meals and transport) |
| **(iv) HIV counselling and testing** |  |  |  |
| Supplies (test kits, pricking needles, gloves, alcohol swabs, sharps containers, plastic waste bags) | ✔ | ✔ | ✔ |
| Personnel (lay counsellors, nurses) | ✔ (100% lay counsellors) | ✔ (12% time community health workers) | ✔ (100% community health workers)  (Sometimes professional nurses do HCT at specified % time) |
| Field materials (counsellor work t-shirts, umbrellas) | ✔ | ✗ | ✗ |
| Stationary, mobile airtime vouchers for data collection | ✔ (100% stationary, 30% mobile airtime vouchers) | ✔ Stationary | ✔ Stationary |
| Cellphone purchases | ✔ | ✗ | ✗ |
| Dry blood spots (DBS) for lab tests (quality assurance) | ✔ (cost of 5% of samples) | ✗ | ✗ |
| **(v) Lay counsellor supervision** |  |  |  |
| Personnel (site project manager, lay counsellor supervisors, drivers) | ✔ (all 100%) | ✗ | ✗ |
| Personnel (professional nurse) | ✗ | ✔ (12% professional nurse team leader) | ✔ (professional nurse at specified % time) |
| Vehicles (vehicle maintenance, insurance and fuel, & transport re-imbursements) | ✔ (90%) | ✔ (12% cost based on time effort) | ✗ |
| Capital - Office equipment (phone, printer, copier), supplies and furniture | ✔ (80%) | ✔ (12% time) | ✔ |
| Data console monthly hosting | ✔ (30 %) | ✗ | ✗ |

✗ (input excluded), ✔ (input included)

**Box 1: Operational scenario**

Good Start HBHCT was implemented as a vertical programme trial in a research context. A number of activities would vary if implemented in a non-research structure, for example in a district level health system. Total costs of the project may therefore be lower. Resources were shared between intervention and research activities, and in other instances, resources were intensified in the intervention. For example, supervision intensity, drivers, the management team, start up costs, community mobilization activities, and t-shirts given to tested clients. Inputs that would be relevant in the operational scenario are varied and presented in Table 2. All research costs were excluded. Further, the operational scenario would be integrated into community outreach programmes already existing in South Africa and implemented by the department of health. Thus, the design of the operational scenario is also informed by the PHC re-engineering ward based outreach model for South Africa^28 29^. In the South African PHC outreach team structure, 1 professional nurse supervisor is responsible for 6 CHWs who work in a ward (village with a catchment population of approximately 7660 people or 1619 households) with each CHW responsible for 270 households on average^28 29^. We varied lay counsellor salaries since CHWs in the outreach team are paid stipends worth about US$100.

Costs of activities that would not be repeated if the intervention were to be integrated into existing community programs at a district level were excluded. These included costs of designing the intervention, developing training manuals and study tools which were one off activities. Office space was included even though outreach teams are community based. It is important that there is designated office space for the team for meetings, filing and other activities in the clinic. Vehicle maintenance, fuel, and stationary inputs were included and apportioned for their time use.

Personnel were varied to acknowledge differences in salaries, skills and type of management between personnel employed in the intervention and the district level staffing. An equivalent of a site project manager would be the facility manager (at 5% time) who manages outreach teams. A level above this person is the district/sub-district manager, thus included in the operational scenario. However, this person is likely to spend very little time in the community HCT activities as they have numerous other roles. We hypothesized the district manager time to be 1%.

An equivalent of supervisors would be professional nurses (team leaders who supervise CHWs) and their salary equivalent was used. Drivers were excluded since professional nurse supervisors drive themselves. However, vehicles were included since they would still be needed for CHW field supervision.

Since CHWs in outreach teams have other roles, we estimated HCT to take up about 12% of their time based on our intervention data where lay counsellors took on average 30 minutes to test and counsel 1 client. If these CHWs work an 8-hour day, they could schedule to test at least 2 clients a day, which translates to 1 hour of their 8-hour working day although this does not take into account the time taken moving between households. We included 12% of the lay counsellor salaries based on this assumption. Similarly, vehicle costs, relevant overheads and professional nurse supervisor time were allocated 12% based on CHW time spent on HCT.

Also, dry blood spot (DBS) testing was a project activity for quality assurance and would not necessarily be part of a scaled up programme.

**Table B: Sensitivity analysis**

|  | **Average cost per client (HBHCT), US$** | **Average cost per client (Clinic), US$** | **ICER, US$** |
| --- | --- | --- | --- |
| **Baseline (US$)** | **29** | **38** | **19** |
| **Discount rate** | | | |
| 3% | 28 | 37 | 20 |
| 6% | 28 | 37.8 | 20 |
| *9% | 29 | 38 | 19 |
| **†Nurse salaries (US$)** | | | |
| 20 464.52 |  | 37.16 |  |
| *25 640.00 |  | 38 |  |
| 30 815.07 |  | 39.63 |  |
| **†Test kits confirmatory (SENSA and determine)** | | | |
| *58.08 | 28.54 | 38.40 |  |
| 115.07 | 28.57 | 39.16 |  |
| **†Test kits screening (SD-bioline and G-ocean)** | | | |
| *30.68 | 28.54 | 38.40 |  |
| 31.64 | 28.54 | 38.45 |  |
| **Catchment population** |  |  |  |
| 22 099 | 28.54 | 41.46 | 19 |
| 23 864 | 26.41 | 38.45 | 17 |

*Estimate used in main analysis, † ICER = $19 at all levels of the variable
